# Mapping knowledge structure and emerging trends in non-invasive brain-computer interface for stroke rehabilitation

**DOI:** 10.1016/j.ibneur.2026.04.011

**Published:** 2026-04-24

**Authors:** Ying Li, Jiaying Chen, Yu Wang, Jinghui Huang, Fanfu Fang

**Affiliations:** Department of Rehabilitation Medicine, The First Affiliated Hospital of Naval Medical University, Shanghai, China

**Keywords:** Stroke, Brain–computer interface, Bibliometrics, Rehabilitation, Neurotechnology

## Abstract

**Objective:**

To explore the current research landscape and emerging frontiers in the application of non‑invasive brain–computer interface (BCI) technology in the field of stroke.

**Methods:**

Publications related to non‑invasive BCI technology in stroke were retrieved from the Web of Science Core Collection database between January 2014 and March 2025. Only English articles and reviews were included; conference papers, editorials, and corrections were excluded.Bibliometric software was employed to construct visual knowledge maps based on authors, institutions, keywords, and other metrics.

**Results:**

After excluding items such as publisher corrections, editorial materials, and conference papers, 587 publications were included. Over the past decade, the annual number of publications showed an upward trend. China (177 publications) contributed the highest volume of output globally. The most prolific author was Jochumsen, Mads (17 publications), and Aalborg University (31 publications) was the leading institution. The journal with the highest number of publications was IEEE Transactions on Neural Systems and Rehabilitation Engineering(60 articles), while the Journal of Neural Engineeringreceived the most citations (2129). Keyword analysis and burst detection revealed that research hotspots mainly focus on signal acquisition methods, EEG‑based signal types, neural mechanisms, algorithms, external devices, and their impact on functional rehabilitation after stroke.

**Conclusion:**

Over the past ten years, advances in technology and interdisciplinary collaboration between medicine and engineering have provided new opportunities for stroke rehabilitation through non‑invasive BCI. This technology shows great clinical value in promoting neural plasticity and functional recovery in stroke patients.It is projected that future research will emphasize multimodal integration, innovations in algorithms such as deep learning, and breakthroughs in material technology, which are expected to represent major research directions and hotspots in the field.

## Highlights

• First comprehensive bibliometric analysis mapping non-invasive BCI research in stroke rehabilitation.
• Identifies and compares dominant paradigms: BCI-robotics, BCI-FES, and BCI-VR.
• Reveals a field shift from single-modal systems to multi-modal platforms for synergistic effects.
• Evidence suggests BCI-FES may outperform BCI-robotics for upper limb recovery.
• Outlines frontiers like BCI-neuromodulation to guide future clinical and translational research.

## 1 Introduction

As a major global public health challenge, stroke is characterized by high incidence, mortality, disability rates, and substantial socioeconomic burden. Post-stroke patients often experience impairments such as dysfunctional motor cortex reorganization (affecting motor function), damage to language centers (affecting speech function), and cognitive deficits (Cheng et al., 2024). Although conventional rehabilitation approaches can partially restore patient function, their capacity to modulate the underlying mechanisms of neural plasticity remains limited. In recent years, brain–computer interface (BCI) technology has emerged as a promising avenue for post-stroke functional recovery. By enabling real-time acquisition, decoding, and feedback of neural signals, BCI facilitates bidirectional interaction between the brain and external devices, offering novel pathways for neuromodulation (Liu et al., 2025).

A BCI is a communication system that directly connects the brain to a computer or external device without relying on muscular or peripheral nerve pathways. It operates by extracting and interpreting central nervous system signals (e.g., electrical or magnetic activity) via specialized equipment, thereby allowing users to control external assistive devices—such as computers, robotic aids, virtual keyboards, or functional electrical stimulators—through direct brain–machine interaction. This process promotes the remodeling of neural circuits and improves functional outcomes (Liu et al., 2025, Chen et al., 2024). Based on the degree of invasiveness, BCIs can be categorized as invasive, semi-invasive (He et al., 2024), or non-invasive. Non-invasive BCI systems acquire brain signals using external sensors placed on the scalp or through non-invasive external devices. Common signal modalities include electroencephalogram (EEG), magnetoencephalogram (MEG), functional magnetic resonance imaging (fMRI), and functional near-infrared spectroscopy (fNIRS). Owing to their safety, non-invasiveness, portability, and cost-effectiveness, non-invasive BCIs have been widely adopted in clinical stroke rehabilitation and demonstrate considerable potential (Casimo et al., 2017).

Although existing literature has addressed advances in BCI technology for rehabilitation, there remains a lack of comprehensive and objective analysis focusing specifically on non-invasive BCI applications in stroke recovery. To address this gap, this study conducts a bibliometric analysis of relevant publications indexed in the Web of Science (WOS) core collection. Using scientometric tools, we construct knowledge maps to provide an overview of the research landscape in terms of country, institution, author, and keyword distributions. This approach allows us to elucidate the intellectual structure and developmental trajectory of the field, while visually highlighting research hotspots and emerging trends.

## 2 Materials and methods

**Fig. 1:**
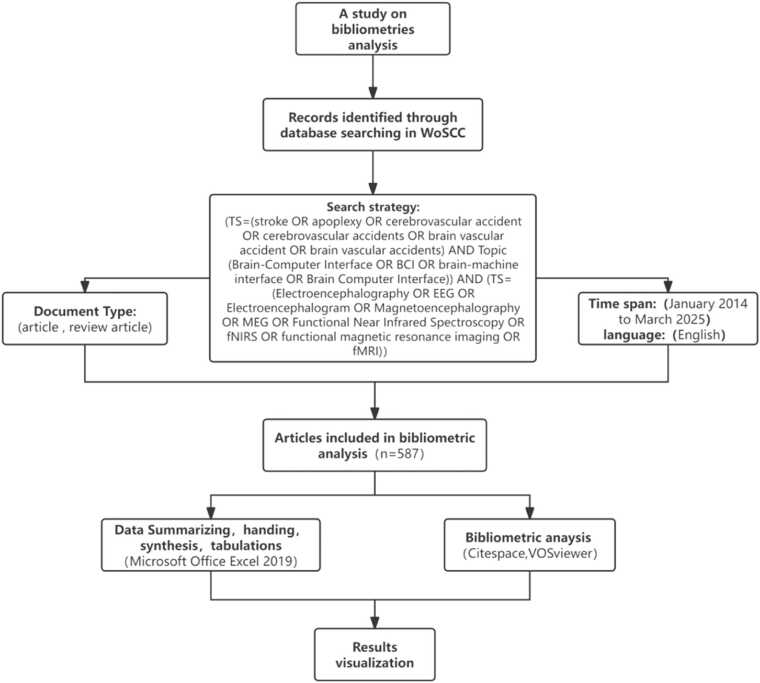
Workflow flowchart of the bibliometric analysis in this study.

Fig. 1 presents the workflow flowchart of this bibliometric study, illustrating the complete process including literature retrieval, screening, inclusion/exclusion criteria, duplicate removal, and bibliometric analysis.

### 2.1 Data Sources

The literature data were retrieved from the Web of Science (WOS) Core Collection, including the SCI-EXPANDED, CCR-EXPANDED, and IC sub-databases. The search strategy was as follows: Topic (stroke OR apoplexy OR cerebrovascular accident OR cerebrovascular accidents OR brain vascular accident OR brain vascular accidents) AND Topic (Brain-Computer Interface OR BCI OR brain-machine interface OR Brain Computer Interface) AND Topic (Electroencephalography OR EEG OR Electroencephalogram OR Magnetoencephalography OR MEG OR Functional Near Infrared Spectroscopy OR fNIRS OR functional magnetic resonance imaging OR fMRI). The time span was set from January 2014 to March 2025, and the language was limited to English. Document types were confined to articles and reviews. Using the Duplicates Removal function in CiteSpace 6.2.R3, items such as publisher corrections, editorial materials, conference papers, and retractions were removed. A total of 587 relevant records were identified in WOS. Two evaluators independently screened the titles and abstracts of the literature, and no irrelevant publications were excluded, resulting in the final inclusion of 587 publications. The data were exported in plain text format, renamed and saved in the form of "download_1", and imported into CiteSpace 6.2.R3 software.

### 2.2 Methods

CiteSpace and VOSviewer are visualization software tools designed for analyzing scientific literature, constructing knowledge maps, and examining the current status, hotspots, frontiers, and trends within a research field. They reflect the frequency and strength of connections among research objects through node size and link thickness. Using CiteSpace or VOSviewer, relevant parameters were configured as follows: the time span was set from January 2014 to March 2025; term sources and key pathways were set to system defaults; and visual network analyses were conducted for regions, institutions, journals, authors, co-cited references, keywords, burst terms, and keyword clustering, respectively.

## 3 Results

### 3.1 Publication trends

**Fig. 2:**
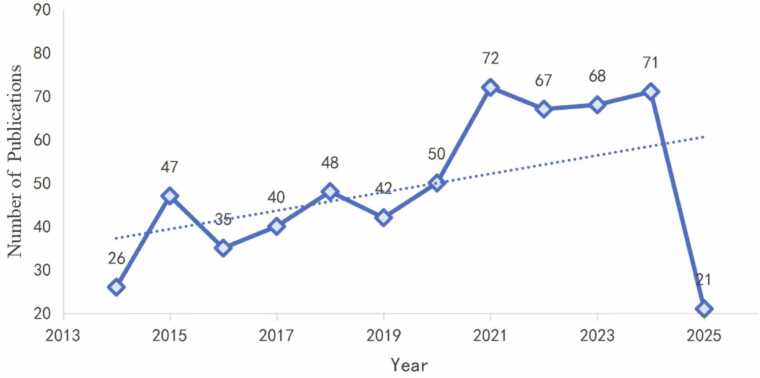
Number of publications related to non-invasive BCI in field of stroke rehabilitation from 2014 to 2025.

Over the past decade, the annual number of publications has generally shown an upward trend (Fig. 2). After reaching 72 publications in 2021, the annual output remained relatively stable from 2021 to 2024. Based on the overall growth trend observed in the line graph, it is projected that this research field will continue to grow steadily in the coming years. As of March 2025, when this article was prepared, 21 publications had been recorded for the year, resulting in a cumulative total of 587 publications.

### 3.2 Countries/regions, institutions, and journals

**Fig. 3:**
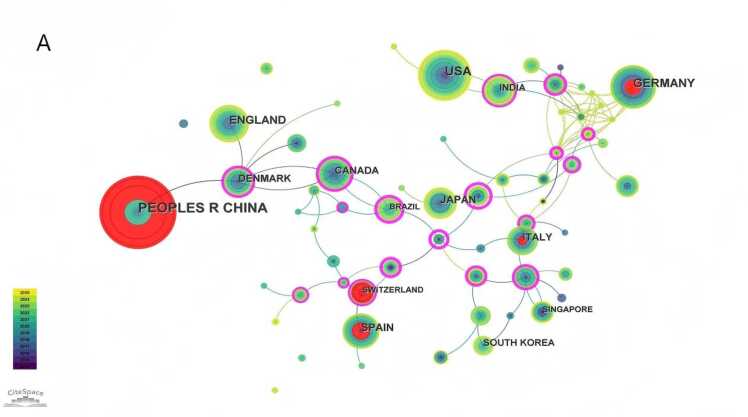

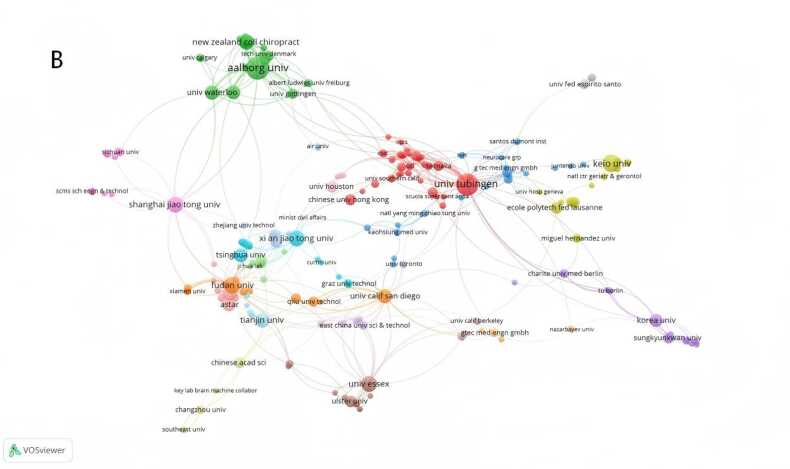

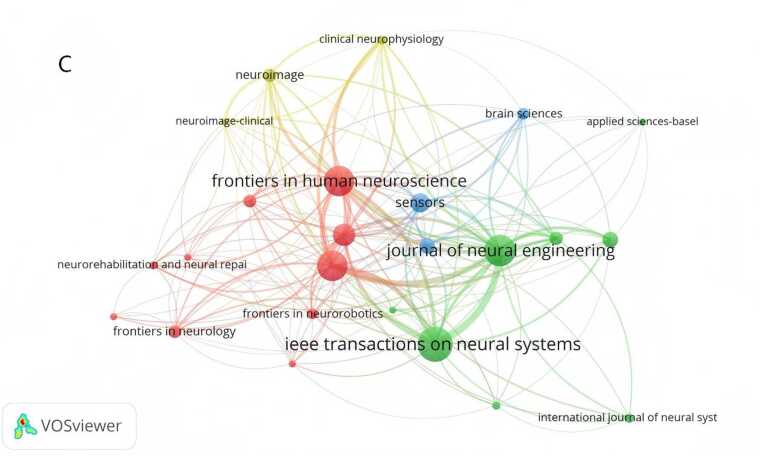
A International collaboration network map related to non-invasive BCI in field of stroke rehabilitation from 2014 to 2025.（Node size represents the number of publications in the corresponding country/region. Node color indicates the average publication year of the included literature. Lines between nodes reflect collaborative connections among different countries/regions. The purple circle represents relatively high centrality of the node. Red nodes denote a significant burst of publications in the recent short term.）. B Institutional collaboration network map related to non-invasive BCI in field of stroke rehabilitation from 2014 to 2025.（Node size represents the number of publications in the corresponding institution. Node color indicates the average publication year of the included literature. Lines between nodes reflect collaborative connections among different institutions.）. C Journal collaboration network map related to non-invasive BCI in field of stroke rehabilitation from 2014 to 2025.（Node size represents the number of publications in the corresponding journal. Node color indicates the average publication year of the included literature. Lines between nodes reflect collaborative connections among different journals.）.

A collaborative network map of countries/regions was generated using CiteSpace (Fig. 3A). Over the past decade, the top five countries/regions by publication volume in the field of non-invasive BCIs for stroke were China (177 publications), the United States (91 publications), Germany (71 publications), the United Kingdom (58 publications), and Japan (45 publications), with China leading by a significant margin. In Fig. 3A, the nodes for China, Germany, Switzerland, and Spain are highlighted in red, indicating a high burst of publications in a short period. The purple ring denotes centrality, which reflects influence in terms of information dissemination within the field; Belgium ranked highest in centrality (0.92). An institutional collaboration network was constructed using VOSviewer (Fig. 3B). The top ten publishing institutions were predominantly universities, with Aalborg University (Denmark, 31 publications) and the University of Tübingen (Germany, 29 publications) being the most productive. Four Chinese institutions were among the top ten: Fudan University (16 publications), Shanghai Jiao Tong University (15 publications), Xi'an Jiaotong University (15 publications), and Tianjin University (13 publications) (see Table 1). Journal analysis via VOSviewer identified 24 journals that had published five or more articles in this field. The most prolific journal was *IEEE Transactions on Neural Systems and Rehabilitation Engineering*(60 publications), while the *Journal of Neural Engineering*had the highest total citation count (2129 citations), both demonstrating significant influence in the field (Fig. 3C).

**Table 1:** Top 10 institutions in the field of stroke with the number of publications on non-invasive BCI technology from January 2014 to March 2025.

**Ranking**	**Institution**	**Publication Count**	**Citation Count**
1	Aalborg University	31	998
2	University of Tübingen	29	1241
3	Keio University	18	367
4	Fudan University	16	289
5	University of Essex	15	613
6	Shanghai Jiao Tong University	15	454
7	Xi'an Jiaotong University	15	263
8	New Zealand College of Chiropractic	14	347
9	Nanyang Technological University	14	764
10	Tianjin University	13	221

### 3.3 Analysis of contributing authors

**Fig. 4:**
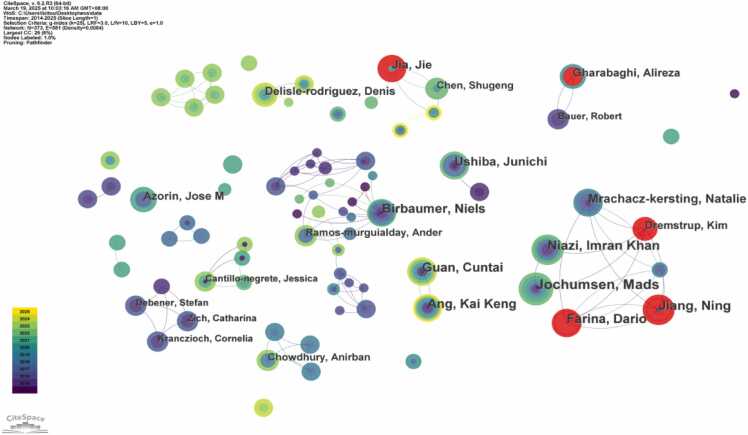
Author collaboration network map related to non-invasive BCI in field of stroke rehabilitation from 2014 to 2025.（Node size represents the number of publications in the corresponding author. Node color indicates the average publication year of the included literature. Lines between nodes reflect collaborative connections among different authors. Red nodes denote a significant burst of publications in the recent short term.）.

An analysis of contributing authors was conducted using CiteSpace. The top three authors by publication volume were Jochumsen, Mads (14 publications), Niazi, Imran Khan (12 publications), and Jiang, Ning (12 publications). Among the top ten most prolific authors, only one was from China: Jia, Jie (9 publications) from Huashan Hospital, Fudan University. In Fig. 4, the nodes for authors Jiang, Ning, Farina, Dario, Jia, Jie, Dremstrup, Kim, and Gharabaghi, Alireza are highlighted in red, indicating a concentrated period of high research activity and a burst of publications within a short timeframe. Furthermore, the authors demonstrate a clustered collaboration pattern, reflecting strong cooperative networks within the field.

### 3.4 Keyword co-occurrence, clustering, and burst

**Fig. 5:**
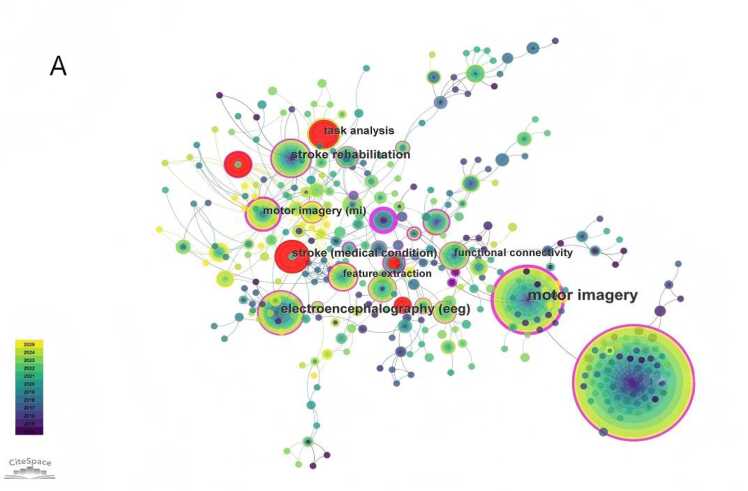

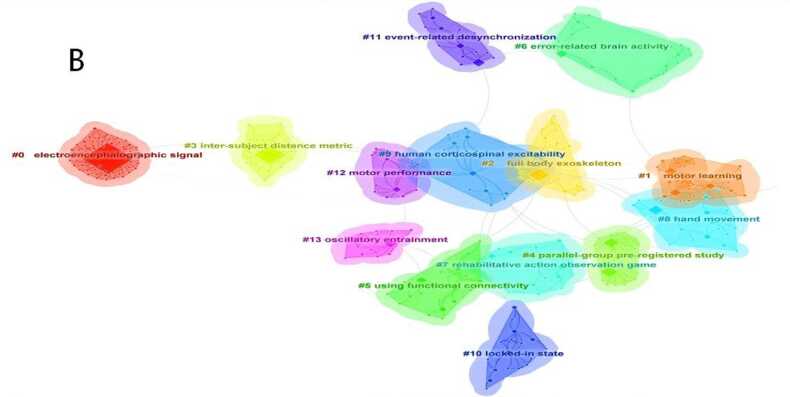

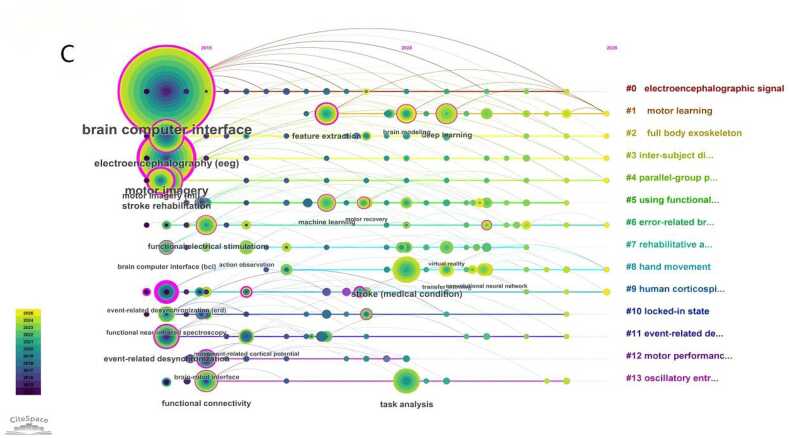
A Keyword co-occurrence network diagram related to non-invasive BCI in the field of stroke rehabilitation from 2014 to 2025.（Node size represents the number of publications in the corresponding Keyword. Node color indicates the average publication year of the included literature. Lines between nodes reflect collaborative connections among different Keywords. The purple circle represents relatively high centrality of the node. Red nodes denote a significant burst of publications in the recent short term.）. B Keyword clustering diagram related to non-invasive BCI in field of stroke rehabilitation from 2014 to 2025.（Different colored blocks represent distinct clusters. The size of each colored block reflects the number of keywords and association strength within the cluster; a larger block indicates more concentrated or active research on the corresponding topic. Overlaps between colored blocks suggest close correlations among different clusters, meaning that they share partial common keywords.）. C Keyword clustering timeline diagram related to non-invasive BCI in field of stroke rehabilitation from 2014 to 2025.（The horizontal axis represents the publication year, while the vertical axis lists the keyword clusters. Each circle represents a keyword; its size reflects the frequency of occurrence, and its color indicates the average publication year of related literature. The lines show the evolutionary relationships and connections between keywords over time, illustrating the temporal development of research themes in the field.）.

DetectionA keyword co-occurrence network was generated with keywords as nodes (Fig. 5A). The top 10 keywords by frequency were: *brain-computer interface*, *motor imagery*, *electroencephalography (EEG)*, *stroke rehabilitation*, *stroke*(medical condition), *task analysis*, *functional connectivity*, *feature extraction*, *event-related desynchronization*, and *functional electrical stimulation*. Using CiteSpace's log-likelihood ratio algorithm, a keyword clustering map was produced (Fig. 5B), identifying the following major clusters: #0 "eeg signal", #1 "motor learning", #2 "whole-body exoskeleton", #3 "cross-individual distance metric", #4 "parallel group pre-registration study", #5 "using functional connectivity", #6 "error-related brain activity", #7 "rehabilitation action observation game", #8 "hand movement", #9 "human corticospinal excitability", #10 "locked-in state", #11 "event-related desynchronization", #12 "motor performance", and #13 "oscillatory synchronization". Among them, #0 "eeg signal" is the most common non-invasive neural signal modality for clinical BCI rehabilitation, providing stable and real-time data support for motor intention decoding. #1 "motor learning" corresponds to the core neuroplasticity mechanism of post-stroke motor function recovery. #2 "whole-body exoskeleton" represents a typical external assistive device widely used in clinical practice to assist limb movement and promote rehabilitation training.

**Fig. 6:**
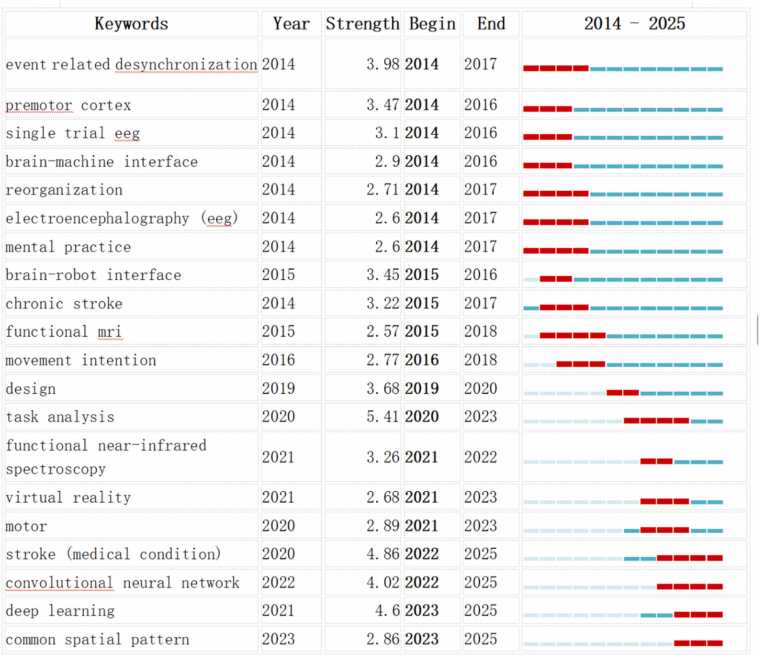
Top 25 keywords with the strongest citation bursts related to non-invasive BCI in the field of stroke rehabilitation from 2014 to 2025. （Blue lines indicate time intervals. The left endpoint of each segment represents the start time of the keyword, and the right endpoint represents the end time. Light blue denotes periods when the keyword did not appear; dark blue denotes periods when the keyword appeared; red denotes periods when the keyword showed a strong citation burst.）.

Following the clustering analysis, a keyword cluster timeline map was created (Fig. 5C), with the publication year and cluster labels on the X and Y axes, respectively, illustrating the temporal evolution of these research themes. Fig. 6 displays the top 20 keywords with the strongest citation bursts. The red segments indicate the duration of each burst, with the start and end points marking the burst period. Notable keywords that experienced significant bursts include *event-related desynchronization*, *premotor cortex*, *single-trial eeg*, *brain-computer interface*, *reorganization*, *eeg*, *mental practice*, *brain-robot interface*, *chronic stroke*, *functional mri*, *motor intention*, *task analysis*, *functional near-infrared spectroscopy*, *virtual reality*, *movement*, *stroke*(medical condition), *convolutional neural network*, *deep learning*, and *common spatial pattern*, representing prominent research trends during their respective periods.

Keywords, which encapsulate the core content of articles, provide a direct and sensitive means of identifying research hotspots and trends in the application of non-invasive BCI for stroke through co-occurrence, clustering, and burst analyses. Categorizing the results of these analyses reveals that the primary focuses can be classified into the following aspects. The main focus lies on the mechanisms of action of non-invasive BCI technology in the stroke field, including: the signal acquisition modalities (electroencephalography, functional near-infrared spectroscopy, functional magnetic resonance imaging); EEG-based signal types (event-related desynchronization, error-related brain activity, oscillatory synchronization); involved neural mechanisms (motor imagery, motor learning, premotor cortex, functional connectivity, reorganization, human corticospinal excitability); and associated computer algorithms (feature extraction, convolutional neural network, deep learning, common spatial pattern). Beyond the mechanistic studies, explorations also include the integration of non-invasive BCI with external devices (whole-body exoskeleton, brain-robot interface, functional electrical stimulation, virtual reality) and its functional impact on stroke patients (movement, hand movement, motor performance).

### 3.5 Co-cited references

**Fig. 7:**
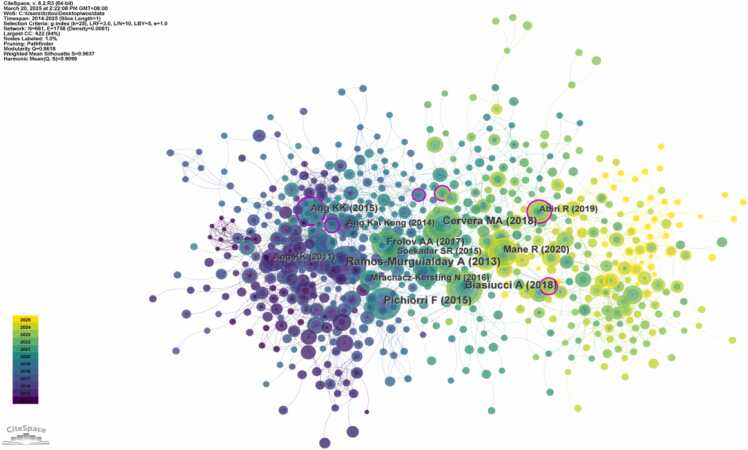
Co-citation network map related to non-invasive BCI in field of stroke rehabilitation from 2014 to 2025.（Node size represents the number of publications in the corresponding Co-citation. Node color indicates the average publication year of the included literature. Lines between nodes reflect collaborative connections among different Co-citation.）.

As shown in Fig. 7, a co-citation network map was generated by selecting "Reference" as the node type. Analysis of highly co-cited references helps clarify the knowledge base of hot research areas. The citation frequency of a publication serves as an indicator of its academic significance and influence. Table 2 lists the top five most co-cited references in the field of non-invasive BCI for stroke. The most frequently co-cited study was a 2013 controlled trial (Ramos-Murguialday et al., 2013). This seminal work demonstrated that integrating BCI training into goal-oriented physical therapy could induce motor function improvements in chronic stroke patients without residual finger movement, suggesting that BCI may open new avenues for neurorehabilitation after stroke.

**Table 2:** Top 5 highly cited articles related to noninvasive BCI in the field of stroke.

Ranking	Title	First Author	Frequency	Quartile (Impact Factor)	Year
1	Brain-machine interface in chronic stroke rehabilitation: a controlled study	Ramos-Murguialday A	87	Q1（8.1）	2013
2	Brain-actuated functional electrical stimulation elicits lasting arm motor recovery after stroke	Biasiucci A	68	Q1（14.7）	2018
3	Brain-computer interfaces for post-stroke motor rehabilitation: a meta-analysis	Cervera MA	66	Q1（4.4）	2018
4	Brain-computer interface boosts motor imagery practice during stroke recovery	Pichiorri F	61	Q1（8.1）	2015
5	BCI for stroke rehabilitation: motor and beyond	Mane R	48	Q2（3.7）	2020

## 4 Discussion

A substantial body of literature has established a foundation for exploring non-invasive BCI technology in the field of stroke. Based on the results of keyword co-occurrence, clustering, and high-frequency burst detection analyses, the research landscape of non-invasive BCI for stroke can be broadly categorized into the following four directions for discussion: (1) mechanisms of action (acquisition modalities, EEG-based BCI systems); (2) functional rehabilitation in stroke (motor, cognitive, affective, speech); (3) external feedback devices (robotics, functional electrical stimulation, virtual reality, non-invasive brain stimulation); and (4) research hotspots and frontiers (multimodal BCIs, algorithms, materials).

### 4.1 Mechanisms of action

Non-invasive BCIs translate recorded brain activity into control signals for external devices, offering a promising approach for post-stroke motor recovery. This process relies on decoding user intent from non-invasively acquired neural signals to establish closed-loop feedback, which is hypothesized to promote functional restoration through neuroplasticity.Several non-invasive neuroimaging techniques support BCI operation.EEG is most common in clinical applications due to its high temporal resolution, despite limited spatial precision. fNIRS offers a portable alternative, measuring cortical hemodynamic changes, while MEG and fMRI provide superior spatial resolution at the cost of practicality for routine use (Naseer and Hong, 2015). Auxiliary technologies such as eye tracking and electromyography are often integrated (Mang et al., 2023, Dillen et al., 2024a), while emerging approaches like ultrasound-based methods have also been proposed (Gan et al., 2023).

In stroke rehabilitation, EEG-based BCIs typically decode movement intention from patterns such as sensorimotor rhythms (μ, β), event-related potentials (P300), or steady-state evoked potentials (SSVEP) (Abiri et al., 2019, Elashmawi et al., 2024, Ma et al., 2024, Kaviri and Vinjamuri, 2024). Machine learning and deep learning algorithms translate these signals into commands for robotic aids or sensory feedback, creating a continuous central-peripheral-central loop that reinforces targeted neural circuits (Li et al., 2023, Jia, 2022).

BCI training paradigms commonly engage the motor system through three approaches: Motor Imagery (mental simulation of movement), attempted Movement Execution (effortful intention to move), and Action Observation (viewing biological motion) (Liu et al., 2025). By activating shared motor networks, these paradigms provide targeted stimulation to induce use-dependent plasticity and facilitate the recovery of motor function (Yue et al., 2023).

### 4.2 Functional rehabilitation after stroke

Non-invasive BCI technology shows growing potential for functional rehabilitation after stroke, particularly in motor, cognitive, affective, and language domains.

3.2.1 Motor Function Rehabilitation Motor recovery is a primary focus of BCI applications in stroke. While conventional therapies like physical training and constraint-induced movement therapy require residual motor ability (Kwakkel et al., 2015), BCI systems offer an alternative for patients with severe paralysis (Ang et al., 2014, Liao et al., 2023). By detecting movement intention or motor imagery (MI) from EEG signals, BCI drives external devices—such as functional electrical stimulation (FES), robotic exoskeletons, or virtual reality (VR) feedback—to form a closed-loop "brain–device–feedback" pathway (Fu et al., 2022, Mansour et al., 2022, Biasiucci et al., 2018, Bai et al., 2020, Li et al., 2025, Khan et al., 2023). This facilitates movement rehearsal and reinforces sensorimotor cortex activation, promoting use-dependent neuroplasticity (Mansour et al., 2022). A key mechanism involves Hebbian plasticity, where synchronized activation of cortical and spinal neurons strengthens synaptic connections (Biasiucci et al., 2018). Meta-analyses confirm that BCI training leads to sustained upper-limb improvement, especially when applied during the subacute phase. Combined BCI-FES appears most clinically promising, with optimal intensity ranging from 20 to 90 min daily, 2–5 sessions/week, over 3–4 weeks (Khan et al., 2023).

3.2.2 Cognitive Rehabilitation BCI-based neurofeedback enhances cognitive functions such as attention and working memory by translating specific EEG rhythms (e.g., suppressed θ, enhanced α/β) into audiovisual feedback (T A et al., 2025, Mane et al., 2020, Carelli et al., 2017). Customized BCI games have improved attention in stroke patients with mild cognitive impairment (T A et al., 2025, Chen et al., 2021). Beyond direct neurofeedback,BCI engages cognitive processes via sensorimotor–cognitive interactions: during MI, prefrontal and parietal regions involved in planning and spatial integration are co-activated, enhancing functional connectivity and improving executive functions (Mane et al., 2020, Xu et al., 2023). Integration with VR and eye-tracking further enables immersive, multi-sensory training, boosting spatial orientation and processing speed (Yue et al., 2023, Drigas and Sideraki, 2024, Lee et al., 2022, Gouret et al., 2024).

3.2.3 Affective and Language Rehabilitation Emerging evidence supports BCI’s role in affective regulation (Lorenzetti et al., 2018). Closed-loop neurofeedback systems modulate emotional brain rhythms using music or virtual environments, alleviating post-stroke depression and anxiety (Pino, 2022, Yan et al., 2021). Combined with non-invasive brain stimulation, BCI offers novel interventional pathways for mood disorders (Shanechi, 2019). In language rehabilitation, preliminary studies show that P300-based BCIs can improve verbal outcomes in aphasia (Musso et al., 2022, Kleih and Botrel, 2024). Some systems decode semantic intent or enable letter selection via brain signals and eye-tracking, providing alternative communication channels for individuals with aphasia or locked-in syndrome (Mane et al., 2022, Luo et al., 2022, Tang et al., 2023).Notably, motor, cognitive, affective, and language recovery are interrelated (Mane et al., 2020, Mane et al., 2022). Integrated BCI platforms that leverage AI and multi-modal feedback may holistically enhance stroke rehabilitation, advancing digital therapeutic strategies.

### 4.3 External feedback devices

In stroke rehabilitation, non-invasive BCIs are increasingly combined with robotic devices, functional electrical stimulation (FES), virtual reality (VR), and non-invasive brain stimulation to form integrated intervention systems. These combinations exhibit distinct clinical benefits and mechanistic profiles (Liu et al., 2025, Lima et al., 2024, Qu et al., 2024, Alashram et al., 2022).

BCI-Robotic Systems Robotic devices, such as hand exoskeletons or end-effectors, are commonly coupled with BCIs to support upper limb motor recovery. However, their therapeutic efficacy is influenced by factors including mechanical properties, degrees of freedom, and training protocols, which collectively affect joint mobility, proprioceptive input, and patient compliance (Qu et al., 2024, Veerbeek et al., 2017). Recent advances in soft robotic gloves integrated with multimodal sensors and haptic feedback offer new possibilities for enhancing proprioceptive stimulation and user engagement (Cheng et al., 2020, Zhang et al., 2024).

BCI-FES Integration BCI-FES systems establish a closed-loop "central command–peripheral response" framework that facilitates neural synchronization and promotes motor relearning (Alashram et al., 2022). This combined approach has demonstrated superior and sustained upper-limb functional improvements compared to conventional therapy, benefiting both subacute and chronic stroke patients (Zhang et al., 2023). Meta-analyses suggest that BCI-FES may be more effective than BCI-robotic systems, possibly due to the targeted muscle activation and enriched proprioceptive feedback provided by FES, which strengthens sensorimotor signaling and cortical reorganization (Li et al., 2025).

BCI-VR Interventions By embedding motor tasks within immersive virtual environments, BCI-VR systems enhance motor imagery and cognitive engagement through multisensory feedback, supporting neural reorganization (Maier et al., 2019). Paradigms such as virtual hand manipulation tasks are often used, and real-time difficulty adjustment allows for personalized training (Drigas and Sideraki, 2024). Furthermore, BCI-VR is frequently combined with FES, robotics, or eye-tracking in multimodal setups to integrate visual, auditory, and haptic cues, potentially yielding synergistic effects on proprioception and motor recovery (Drigas and Sideraki, 2024, Ansado et al., 2021, Dillen et al., 2024b).

BCI with Non-Invasive Brain Stimulation Combining BCI with techniques like repetitive transcranial magnetic stimulation (rTMS) or transcranial direct current stimulation (tDCS) aims to modulate cortical excitability and improve signal decoding accuracy (Shu et al., 2018, Ming et al., 2025). For instance, high-frequency rTMS may enhance ipsilesional cortical activity, potentially boosting BCI performance (Shu et al., 2018). However, clinical outcomes remain controversial: a meta-analysis found no significant difference in upper-limb function between BCI-tDCS and control groups, suggesting that parameters such as stimulation protocol, target area, and timing require further optimization and validation through large-scale randomized trials (Lima et al., 2024).

### 4.4 Research hotspots and frontiers

Current neuroimaging tools exhibit inherent limitations in terms of cost, spatiotemporal resolution, and applicable scenarios (Qin et al., 2023). In response, multimodal non-invasive BCI technology is emerging as an innovative frontier. By integrating multi-source data from EEG, fNIRS, fMRI, and eye-tracking, more comprehensive neural activity representation can be constructed, significantly enhancing system accuracy and scenario adaptability. Studies have demonstrated that eye movement-EEG can improve command decoding speed by pre-estimating movement intention (Dillen et al., 2024a, Brickwedde et al., 2022), while EEG-fNIRS fusion systems based on the E-FNet architecture achieved notable accuracies of 95.86% and 95.80% in motor imagery and mental arithmetic tasks, respectively, outperforming single-modality EEG (Yu et al., 2025).

Beyond multimodal BCI systems, future research hotspots also focus on AI-driven closed-loop regulation. Advances in dynamic feature extraction algorithms based on deep learning (e.g., convolutional neural network architectures) may enable millisecond-level adaptive adjustment of neurofeedback parameters and the establishment of personalized rehabilitation models (Elashmawi et al., 2024). Concurrently, ongoing development of novel materials and portable devices is expected to provide crucial support for stable brain signal acquisition and mobile applications (Yuan et al., 2021). These technological pathways collectively reflect the deepening integration of neural engineering and clinical medicine, holding promise for transforming the technological paradigm of stroke diagnosis and treatment.

## 5 Conclusion

This study conducted a bibliometric analysis of the literature on the application of non-invasive BCI technology in the field of stroke, providing researchers with an efficient overview of the research hotspots and trends in this area. Over the past decade, significant progress has been made in non-invasive BCI research for stroke. However, its clinical translation still faces challenges such as insufficient signal stability, considerable individual variability, and prolonged training periods. In addition, most current clinical studies are limited by small sample sizes, short follow-up periods, unclear long-term efficacy, relatively high device costs, and large inter-individual differences, which restrict large-scale clinical application.Future efforts should focus on technological innovations—including novel materials, adaptive algorithms, and artificial intelligence—coupled with interdisciplinary collaboration between medicine and engineering, to advance personalized and precise rehabilitation for stroke patients. The application of non-invasive BCI in stroke holds considerable promise, with multimodal integration, breakthroughs in algorithmic technologies such as deep learning, and the development of new materials likely representing key research directions and future hotspots.Nevertheless, this bibliometric study has several limitations. First, only the Web of Science Core Collection database was retrieved, potentially leading to incomplete literature coverage. Second, Chinese publications and gray literature were not included, which may introduce certain selection bias.

## CRediT authorship contribution statement

**Jiaying Chen:** Writing – original draft, Investigation, Formal analysis, Conceptualization. **Jinghui Huang:** Writing – review & editing, Formal analysis, Data curation, Conceptualization. **Yu Wang:** Writing – original draft, Methodology, Formal analysis, Data curation, Conceptualization. **Ying Li:** Writing – original draft, Software, Formal analysis, Data curation, Conceptualization. **Fanfu Fang:** Writing – review & editing, Visualization, Validation, Methodology, Funding acquisition.

## Consent for publication

The Author confirms: that the work described has not been published before (except in the form of an abstract or as part of a published lecture, review, or thesis); that it is not under consideration for publication elsewhere; that its publication has been approved by all co-authors, if any; that its publication has been approved (tacitly or explicitly) by the responsible authorities at the institution where the work is carried out. The Author transfers to the Founder of the journal the exclusive right to the presented paper, including the right to publish the paper in the English language.The copyright is transferred when the article is accepted for publication.

## Ethics approval and consent to participate

No original clinical data was used in this study, so ethical approval is not required.

## Funding

The conduct of this study was sponsored and funded by National Health Commission of the People's Republic of China (Grant Number SZ2024HL010) and Shanghai Advanced Rehabilitation Medical Technology and Management Innovation Project SHKFJS002F. The funding sources had no role in the study design, execution, or publication.

## Declaration of Competing Interest

The authors declare that they have no known competing financial interests or personal relationships that could have appeared to influence the work reported in this paper.

## Data Availability

The datasets analyzed during the current study are available from the corresponding author upon reasonable request.
